# Supplementary material for: Programming patchy particles for materials assembly design
Source: Proc Natl Acad Sci U S A. 2024 Jun 24;121(27):e2311891121. doi: 10.1073/pnas.2311891121 (PMC11228463; doi:10.1073/pnas.2311891121)
Supplement: Supplementary file 1 — Appendix 01 (PDF) [file pnas.2311891121.sapp.pdf]

PNAS
www.pnas.org

Supporting Information for

Programming patchy particles for materials assembly design

Ella M. King[a,1], **Chrisy Xiyu Du**[b,c,1], **Qian-Ze Zhu**[b], **Samuel S. Schoenholz**[d,e], **Michael P. Brenner**[b,*1]

[a]Department of Physics, Harvard University, Cambridge MA 02139, USA; [b]School of Engineering and Applied Sciences, Harvard University, Cambridge MA 02139, USA; [c]Mechanical Engineering, University of Hawai'i at Mānoa, Honolulu HI 96822, USA; [d]Google Research, Mountainview CA 94043; [e]OpenAI; [1]Ella M. King contributed equally to this work with Chrisy Xiyu Du

Michael P. Brenner.
E-mail:brenner@seas.harvard.edu

This PDF file includes:

Supporting text
Figs. S1 to S6
SI References

Supporting Information Text

1. Implementation

In order to enable differentiable rigid body dynamics, we extend the JAX-MD framework to anisotropic systems. The two major advancements are a new `RigidBody` dataclass and extended integrators with rotational degree of freedom. The `RigidBody` object consists of a `center` and an `orientation`. In the typical case, the `center` stores the information related to translation movements of an object and the `orientation` stores the information related to rotation movements of the object, which may be an array of angles in two dimensions or an array of quaternions in three dimensions.

While the `RigidBody` is most commonly used to store positional information, where the `center` would correspond to the position of the center of mass and the `orientation` would correspond to the rotation of an object based on its initial reference frame, the `RigidBody` class is used to store information about any quantity with both a center of mass and an orientation component. This includes momentum, where the `center` corresponds to the linear momentum and the `orientation` corresponds to the angular momentum, the force, where `center` is linear force and `orientation` is torque, and the mass, where the `center` stores the total mass of the object and the `orientation` stores the moment of inertia tensor. Because `JAX-MD` is built on top of `JAX`, by storing all of these quantities as `RigidBody` objects, we can take advantage of the `tree_map` functionality offered by `JAX` to easily map over these quantities. Among other advantages, this allows us to make use of the differentiability of the code base to compute angular accelerations via gradients rather than via explicit, complex torque expressions (1).

In three dimensions, the `orientation` component of the `RigidBody` object is represented as a quaternion. In order to make energy and force calculations convenient and efficient, we include a set of functions that act on quaternions, including transformations between the body frame and the space frame.

The utility of these functions can be particularly seen in the methods we provide to construct rigid bodies from unions of point particles. We construct a `RigidPointUnion` class that contains a set of positions for the particles that make up the rigid body as defined in the body frame, a set of masses of each constituent particle, an array specifying the number of points in each shape, an array specifying how to index the particle positions according to the shape, and finally species information about the constituent particles. We use the information in the `RigidPointUnion` to construct a pointwise energy function. In essence, the `RigidPointUnion` describes the shape of the rigid body. We note, however, that our code is valid for more general rigid body types, despite the fact that we only provide functionality for rigid bodies composed of point particles. Adding explicit functionality for other types of rigid bodies is an avenue for future work.

In order to run simulations with rigid bodies, we additionally extended the original `JAX-MD` integrators to function with rotational degrees of freedom. We implemented the algorithm developed in (1), which introduces a symplectic integrator that acts on unit quaternions. The algorithm relies on the Trotter-Suzuki operator splitting approach. We note that this method is compatible with both constant energy (NVE) and constant temperature (NVT) ensembles, but is not compatible with constant pressure (NPT) simulations.

We chose to allow for flexibility in our representation of the particles and objects we simulate. Many common Molecular Dynamics (MD) packages use a common representation of objects under the hood, which leads to, for instance, unnecessarily storing orientations for isotropic particles. In order to accommodate varying input types, we make use of the `JAX` `single_dispatch` function. This allows us to use the original `JAX-MD` simulation functions if the input type does not contain rotational degrees of freedom, but to use `RigidBody`-enabled simulation functions if the input type is a `RigidBody`. This allows simulations that do not require functionality for rotational degrees of freedom to maintain higher efficiency. All the code discussed above is now available in the main branch of the `JAX-MD` Github repository (2).

2. Simulation

A. 2D Self-Limited Rings. Here, we investigate the temperature dependence of the yield of trimers and squares in the case of self-limited assembly of squares. We surmised that at a higher temperature, incorrect products would more easily break apart, which would reduce the need for the wider opening angle. Indeed, we find that the optimal opening angle at a higher temperature is much closer to the naively expected 90 degrees. We again compute the yields for triangles, squares, and pentagons for the 90 degree guess and the higher temperature optimal parameters in Fig S5.

Fig. S2 shows the patch interaction evolution as a function of optimization timesteps. We start with randomly generated patch interactions and optimize the interaction matrix alongside of the patch angle. We notice that interaction between different patch species (A-B) is clearly favored to achieve the target finite square morphology, while same species interactions (A-A, B-B) are discouraged. Since we started with random guesses for the patch interactions, A-A and B-B interactions, while small, do not approach zero at the end of the optimization. Judging from the trajectory of interaction evolution, it is clear that patch angle plays a more crucial role in the final assembled structure if the interaction strengths are different enough.

B. 3D Cluster Stabilization. In order to optimize for the stabilization of the 3D octahedron, we begin with particles initialized in an octahedral configuration, but rotated randomly. The particles may resultingly fall apart or may remain in the octahedral configuration, depending on the current set of parameters. Sufficiently long simulations are needed to measure the degree to which the octahedron will stay together, and therefore to provide the gradient calculation with a meaningful signal from the simulation.

To determine the necessary simulation length, we ran forward simulations of the octahedron stabilization and computed the loss as a function of simulation time. These results are shown in Fig. S3B (Yellow line). To reliably observe the relevant

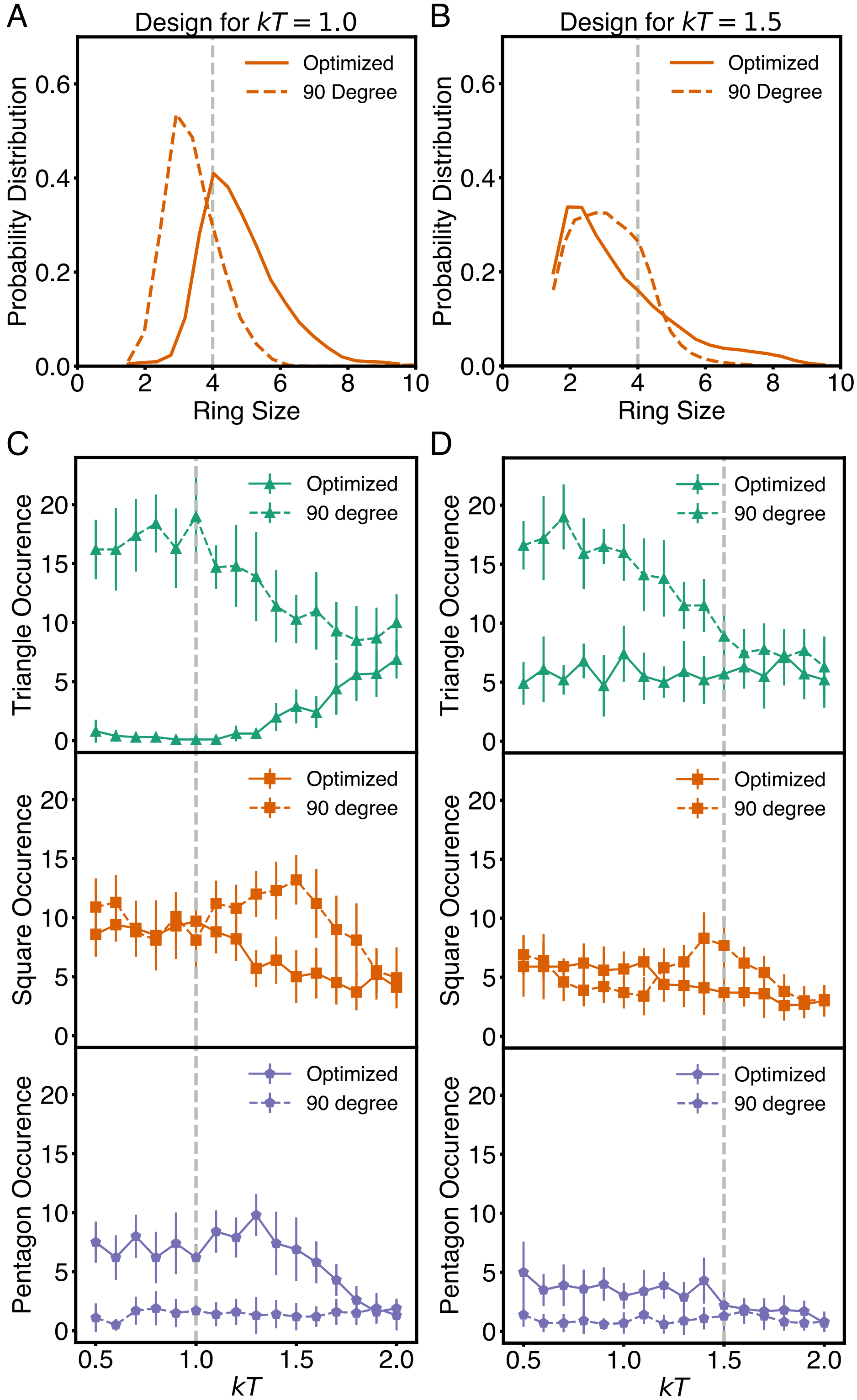

Fig. S1. Optimal square parameters prevent triangle formation. A Yield for our optimal parameters for formation of square rings at kT=1.0 (solid line) and the yield for particles with a 90 degree opening angle (dashed line). B Yield for our optimal parameters for formation of square rings at kT=1.5 (solid line) and the yield for particles with a 90 degree opening angle (dashed line). C Occurance of triangles (incorrect product), squares (correct product) and pentagons (incorrect product) as a function of temperature for our optimal parameters for kT=1.0 (solid line) and for a 90 degree opening angle (dashed line). D Occurance of triangles (incorrect product), squares (correct product) and pentagons (incorrect product) as a function of temperature for our optimal parameters for kT=1.5 (solid line) and for a 90 degree opening angle (dashed line).

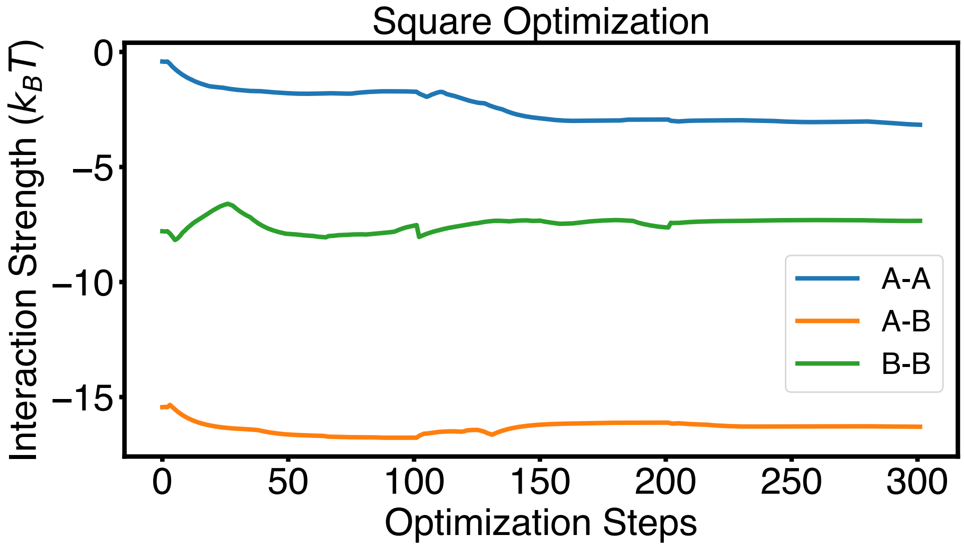

Fig. S2. Patch Interaction Evolution Plot for the patch interactions between same species (A-A and B-B) and different species (A-B) as a function of optimization timesteps. To achieve the finite square morphology, strong A-B interaction is desired while A-A/B-B interactions should be negligible.

changes in the loss function from the initial to the final configuration, we used 200,000 timesteps per simulation. The loss function is described in Fig. S4. The blue curve shows the loss values for the optimal parameters found in our optimization, and the yellow curve shows parameters used in (3) to assemble an octahedron. As expected, because our parameters were optimized to stabilize an octahedron, our parameters show a lower final loss value on average.

3. Optimization

In both the 2D and the 3D examples we discuss, we use a loss function that considers a set of N particles, computes the distances between each particle and its nearest neighbors, and compares these distances to distances computed for a reference structure that also consists of N particles. In the 2D self-limiting assembly case, the reference structure was a 2D trimer or a square. In the 3D case, the reference structure was an octahedron. A graphical representation of this loss function is given in fig S4.

A. Comparison to Literature. As discussed in the main text, we aim to stabilize an octahedral cluster. While we find different optimal parameters than those found by Long et al in (3), this is expected: Long et al aim to assemble an octahedron rather than to stabilize one. We explicitly demonstrate that our optimal parameters are preferred for the task of stabilization in two ways. First, we initialize the optimization from the parameters given by Long et al, and show in Fig S5 that those initial parameters reliably converge to our optimal parameters. Second, we perform forward simulations of stabilization using both our optimal parameters and the parameters found by Long et al. The results are given in Fig S3. We observe that our optimal parameters consistently show a lower loss for stabilization than the parameters given in (3).

References

1. T Miller Iii, et al., Symplectic quaternion scheme for biophysical molecular dynamics. *The J. chemical physics* **116**, 8649–8659 (2002).
2. (https://github.com/jax-md/jax-md) (2023).
3. AW Long, AL Ferguson, Rational design of patchy colloids via landscape engineering. *Mol. Syst. Des. & Eng.* **3**, 49–65 (2018).

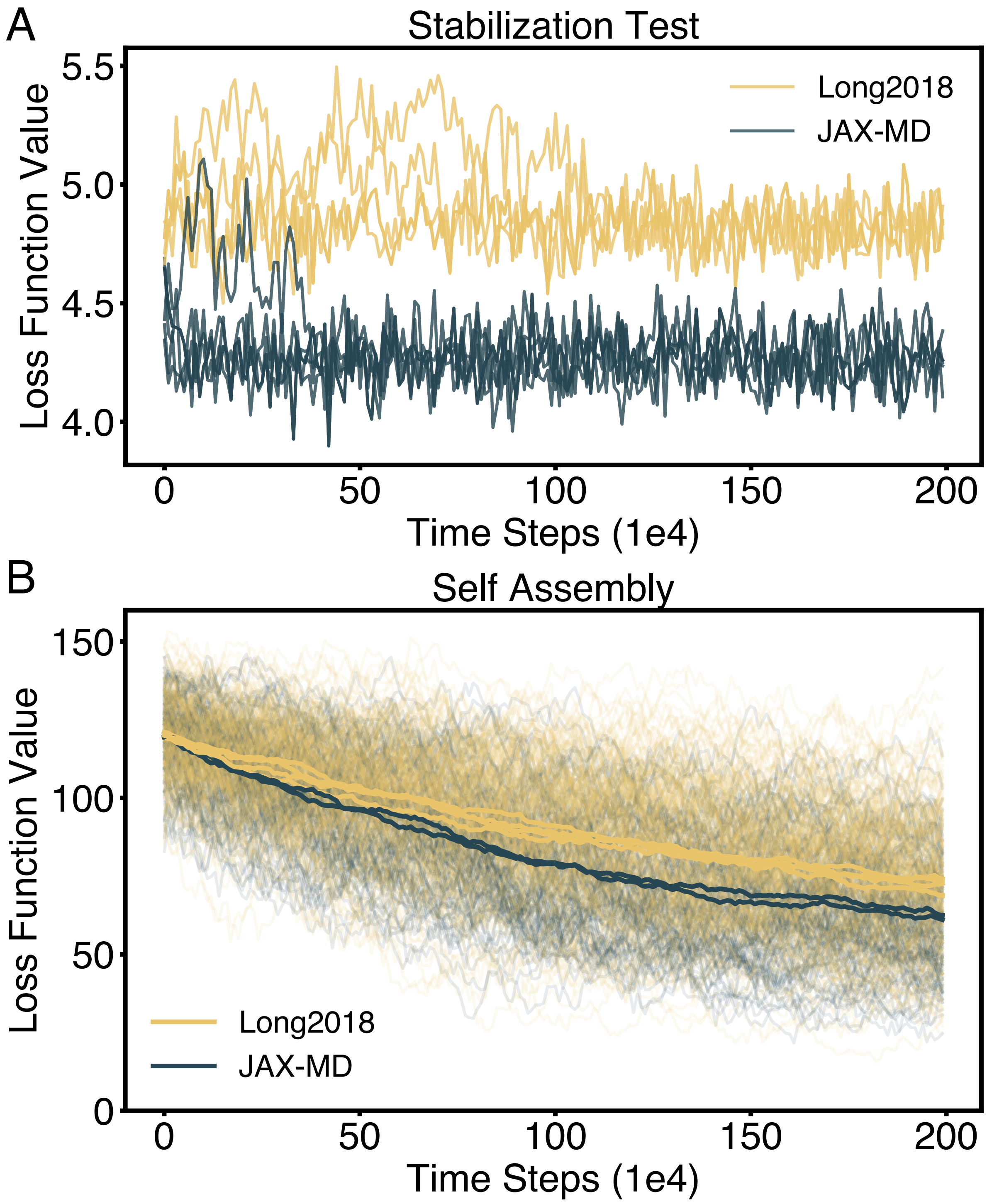

Fig. S3. 3D Cluster: comparsion A Stabilization test between (3) and JAX-MD. B Forward simulation results. In both subfigures, we plot loss as a function of simulation time. Blue curve shows optimal parameters from our optimization, and the yellow curve shows parameters used in (3) to assemble an octahedron.

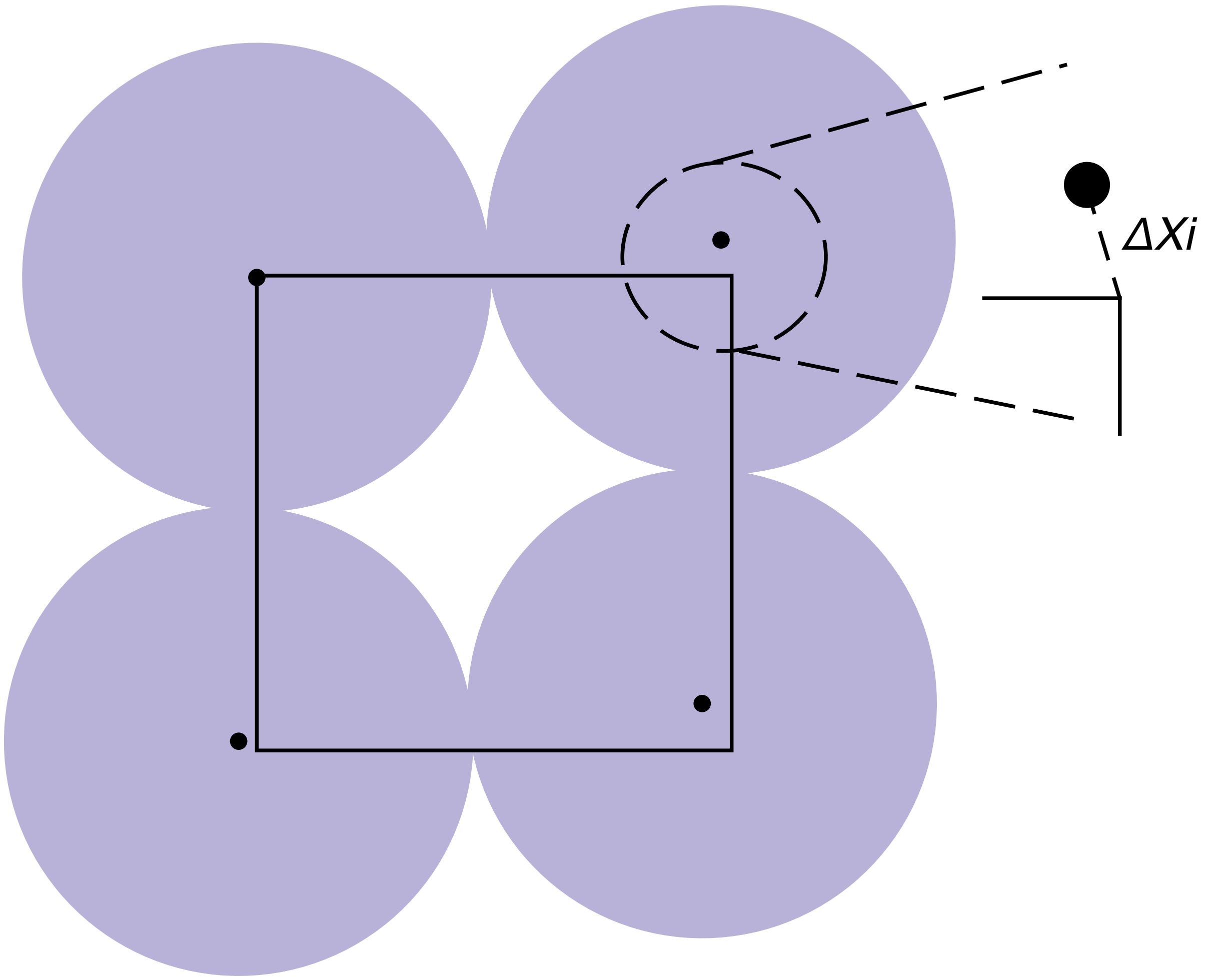

Fig. S4. Graphic illustration of loss function

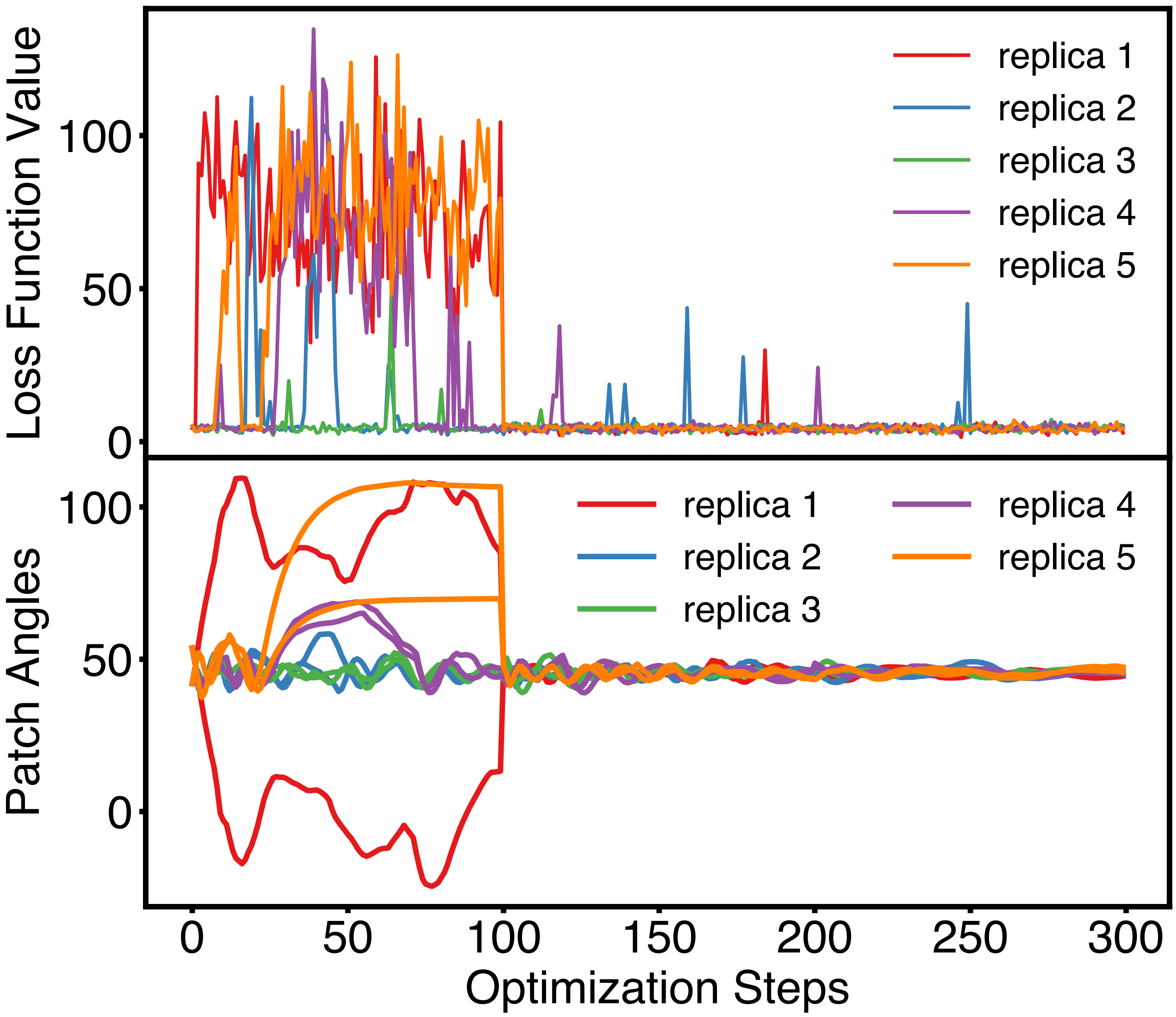

Fig. S5. Stabilization optimization initialized using (3) parameters

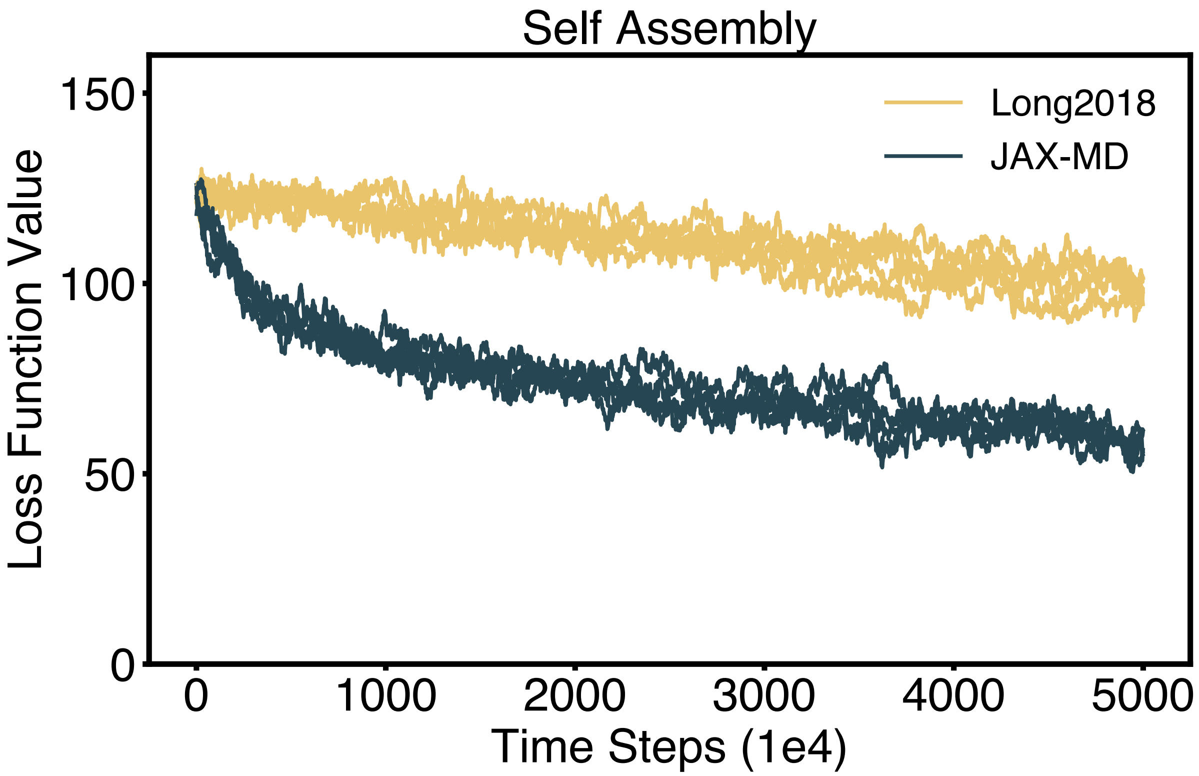

Fig. S6. Larger system ($N = 216$) forward simulations using HOOMD-blue for both (3) parameters and JAX-MD optimized parameters Value of the loss for the optimal parameters over the course of a representative simulation for 2e6 time steps for both literature optimized parameters and `JAX-MD` optimized parameters. `JAX-MD` optimized parameters show a slightly lower loss function value for stabilization test.
